# Time-to-Detection of Inducible Macrolide Resistance in *Mycobacterium abscessus* Subspecies and Its Association with the *Erm*(41) Sequevar

**DOI:** 10.1371/journal.pone.0158723

**Published:** 2016-08-04

**Authors:** Sara Christianson, William Grierson, Daniel Kein, Andrea D. Tyler, Joyce Wolfe, Meenu K. Sharma

**Affiliations:** 1 National Reference Centre for Mycobacteriology, National Microbiology Laboratory Public Health Agency of Canada, 1015 Arlington Street, Winnipeg, MB, Canada, R3E 3R2; 2 Department of Medical Microbiology, University of Manitoba, Room 543–745 Bannatyne Avenue, Winnipeg, Manitoba, Canada, R3E 0J9; Universidad Nacional de la Plata, ARGENTINA

## Abstract

Mutations in the *erm*(41) gene of *M*.*abscessus* group organisms are associated with differences in inducible macrolide resistance, with current recommendations being to hold rapidly growing isolates for up to 14 days in order to ensure that resistance which develops more slowly can be detected. This study aimed to determine the ideal incubation time for accurate identification of inducible macrolide resistance as well as to determine if there was an association between the time taken to detect inducible resistance in *M*.*abscessus* group organisms and their *erm*(41) sequevar. We amplified and sequenced the *erm*(41) genes of a total of 104 *M*.*abscessus* group isolates and determined their sequevars. The isolates were tested for phenotypic clarithromycin resistance at days 7, 10, 14 and 21, using Trek Diagnostics Sensititre RAPMYCO microbroth dilution plates. Associations between *erm*(41) gene sequevars and time to detection of resistance were evaluated using Fisher’s exact test in R. The samples included in this study fell into 14 sequevars, with the majority of samples falling into Sequevar02 (16), Sequevar06 (15), Sequevar08 (7) and Sequvar 15 (31), and several isolates that were in small clusters, or unique. The majority (82.7%) of samples exhibiting inducible macrolide resistance were interpreted as resistant by day 7. Two isolates in Sequevar02, which has a T28C mutation that is associated with sensitivity, showed intermediate resistance at day 14, though the majority (13) were sensitive at day 14. The majority of isolates with inducible macrolide resistance fell into Sequevars 06,08 and 15, none of which contain the T28C mutation. These sequevars were analyzed to determine if there was any correlation between sequevar and time to detection of resistance. None was found. Based on these findings, we recommend the addition of a day 7 read to the CLSI guidelines to improve turn-around-times for these isolates. It is also recommended that *erm*(41) gene sequencing be added to routine phenotypic testing for the resolution of cases with difficult-to-interpret phenotypic results.

## Introduction

*Mycobacterium abscessus* is implicated in many clinically important infections including respiratory infections and skin infections. It is the third most common non-tuberculous mycobacteria (NTM) implicated in respiratory infections, only outnumbered by *M*. *kansasii* and *M*. *avium*.infections [1]. In 2009, Leao et al proposed that *M*. *abscessus* isolates identified via 16s rRNA gene sequencing should be differentiated into 3 subspecies, *M*. *abscessus* subsp. *abscessus*, subsp. *massiliense* and subsp. *bolletii* using differences in their *hsp*65 and *rpo*B gene sequences [2]. After a re-evaluation of that taxonomy, the *M*. *abscessus* subsp. *massiliense* and *M*.*abscessus* subsp. *boletii* subsp. were combined into a single group based on the application of the Bacteriological Code [2]. More recently the combination of these two subspecies is being re-evaluated, with one of the major items of contention being inducible macrolide resistance and therefore potential treatment differences between the three previously proposed subspecies [3–6].

Macrolides are considered a standard tool in the treatment of NTM infections[1]. The erythromycin ribosomal methlylase (*erm*) gene, which is associated with inducible resistance to macrolide antibiotics, has been identified in several clinically relevant rapidly growing mycobacteria (RGM), including the *erm*(41) gene in the *M*. *abscessus* group [7–9]. In order to detect inducible macrolide resistance phenotypically, the Clinical Laboratory Standards Institute (CLSI) M24 A-2 standard suggests a 14 day incubation time for broth microdilution-based macrolide sensitivity testing, but also states that further studies including large numbers of isolates are required to determine the optimal incubation time for interpretation of minimum inhibitory concentration (MIC) for macrolides [10]. Detection of inducible macrolide resistance can be expedited by sequencing the *erm*(41) gene that is present in organisms. Truncated *erm*(41) genes, or T28C mutations in the *erm*(41) gene render the enzyme non-functional and do not result in inducible macrolide resistance [7,11]. The *erm*(41) gene sequence can further be separated into sequevars based on 13 single nucleotide polymorphisms (SNPs) throughout the gene. So far, the T28C SNP is the only SNP in a full length *erm*(41) gene that has been associated with sensitivity. This study aimed to determine the ideal incubation time for the detection of inducible macrolide resistance and establish whether there is a correlation between the length of time it takes to detect inducible macrolide resistance in *M*.*abscessus* group organisms and their *erm*(41) sequevars.

## Methods

### Strains and subspeciation

A total of 104 *M*.*abscessus* subsp.isolates were selected for this study. Strains were identified as being members of the *M*.*abscessus* group using 16s rRNA gene sequencing as previously described [12,13]. Subspeciation of *M*. *abscessus* subsp. *abscessus*, *M*. *abscessus* subsp. *massiliense* and *M*. *abscessus* subsp. *bolletii* was determined by sequencing the *hsp*65 gene as previously described [13]. A group of 22 *M*. *chelonae* isolates, that do not exhibit inducible macrolide resistance was selected for validation of the stability of clarithromycin in the broth microdilution panels.

### Broth microdilution

Isolates were subcultured from frozen stocks in Bactec MGIT media (Becton Dickenson, franklin Lakes, NJ) and then subcultured to Middlebrook 7H11 agar to obtain pure colonies. Isolates were then inoculated into Trek Diagnostics Sensititre RAPMYCO microbroth dilution plates (Thermo Scientific, Cleveland, OH) according to the manufacturer’s directions and incubated for up to 14 day at 31°C. *M*. *chelonae* isolates were incubated for 21 days to validate the stability of clarithromycin after extended incubation. The clarithromycin MIC was recorded at days 7, 10 and 14. Interpretations of sensitive, intermediate or resistant were made according to the CLSI M24-A2 guidelines [10]. Negative control wells were monitored on the panels to detect possible contamination. To determine if there was any advantage to an extended incubation time, a selection of 14/33 *M*. *abscessus* isolates with MICs indicating macrolide susceptibility at day 14, were incubated to 21 days *erm*(41) gene sequencing and analysis:

We amplified the *erm*(41) genes using the primers ermF and erm41 as previously described (Brown Elliot et al). Sequences were analyzed using the BioNumerics 5.1 software package (GeneMaths, Belgium). Nucleotides at the previously described 13 positions for determination of sequevar were concatenated into a short sequence and clusters were calculated in BioNumerics using UPGMA. Sequevars were assigned as previously described [14]. Sequevars that did not fall into the previously published groups were assigned provisional numbering for the purpose of this study. Statistical significance of the association between sequevar and time–to-detection of resistance were calculated using Fisher’s exact test in R (https://www.R-project.org).

## Results

Twenty one *M*. chelonae strains, which lack the mechanism for inducible macrolide resistance (Nash, 2009), were inoculated into panels and incubated for 21 days to evaluate the stability of the clarithromycin in the panel at that extended time point. Clarithromycin MIC values were relatively stable over the 21 day incubation period for 20/21 strains (S1 Table). One strain did achieve an MIC of 8 μg/mL at day 10, but due to a rapid jump in MIC over a 3 day period, this is suspected to be due to contamination caused by exposing the panel to the air. Based on these findings, we concluded that the clarithromycin present in the panel was sufficiently active over the entire 21 day incubation period.

Identifications, macrolide sensitivity interpretations and *erm*(41) sequevars are presented in Table 1. All 20 *M*. *abscessus* subsp. *massiliense* isolates and 2 isolates closely related to *M*. *abscessus* (1 bp difference by 16s rRNA gene sequencing) had a truncated *erm*(41) gene, and were sensitive to clarithromycin. Sixteen (3 *M*. *abscessus* subsp. *bolletii*, 1 close to *M*. *abscessus* and 12 *M*. *abscessus* subsp. *abscessus*) isolates had T28C mutations and belonged to Sequevar02, with 87.5% of those showing sensitivity to clarithromycin after the 14 day incubation period. Additionally, within Sequevar02, 2 *M*. *abscessus* subsp. *abscessus* isolates with T28C mutations intermediate interpretations at day 14 and 1 strain closely related to *M*. *abscessus* (1 bp away by 16s rRNA gene sequencing) had an intermediate interpretation at day 21. Interestingly, the two strains with intermediate interpretations at day 14, along with two other *M*. *abscessus* subsp. *abscessus* isolates with T28C mutations initially showed inducible macrolide resistance. Repeat testing resulted in two isolates having MICs in the sensitive range on day 14, with the other two having intermediate MICs.

**Table 1:** Subspeciation, days to detection of inducible macrolide resistance, *erm*(41) sequence and Sequevar assignment for study strains^1^.

Study Number	Identification	ClarithromycinResistance	*erm*(41) sequevar^2^	G8	T28	G66	A120	T159	G168	A238	G255	G279	A330	T336	T380	C419
2 isolates	*M*. *abscessus* subsp. *abscessus*	inducible (day 7)	Sequevar01	G	T	G	A	T	G	A	G	G	A	T	T	C
ERM029	M. *abscessus* subsp. *abscessus*	intermediate (day 3)	Sequevar01	G	T	G	A	T	G	A	G	G	A	T	T	C
ERM103	M. *abscessus* subsp. *abscessus*	intermediate (day 14)	Sequevar02	G	C	G	A	C	G	G	G	G	C	T	T	C
ERM104	M. *abscessus* subsp. *abscessus*	intermediate (day 14)	Sequevar02	G	C	G	A	C	G	G	G	G	C	T	T	C
ERM093	M. abscessus subsp. unknown	intermediate (day 21)	Sequevar02	G	C	G	A	C	G	G	G	G	C	T	T	C
10 isolates	M. *abscessus* subsp. *abscessus*	sensitive	Sequevar02	G	C	G	A	C	G	G	G	G	C	T	T	C
3 isolates	M. abscessus subsp. bolletii	sensitive	Sequevar02	G	C	G	A	C	G	G	G	G	C	T	T	C
ERM090	M. *abscessus* subsp. *abscessus*	inducible (day 14)	Sequevar06	G	T	G	A	C	G	G	A	T	C	C	T	C
ERM092	M. *abscessus* subsp. *abscessus*	inducible (day 14)	Sequevar06	G	T	G	A	C	G	G	A	T	C	C	T	C
11 isolates	M. abscessus subsp abscessus	inducible (day 7)	Sequevar06	G	T	G	A	C	G	G	A	T	C	C	T	C
ERM038	M. abscessus subsp. abscessus	resistant	Sequevar06	G	T	G	A	C	G	G	A	T	C	C	T	C
ERM046	M. abscessus subsp. abscessus	resistant	Sequevar06	G	T	G	A	C	G	G	A	T	C	C	T	C
ERM075	M. abscessus subsp abscessus	inducible (day 10)	Sequevar07	G	T	G	A	C	G	G	A	G	C	T	T	C
2 isolates	M. abscessus subsp. abscessus	inducible (day 10)	Sequevar08	G	T	G	A	T	C	A	G	G	A	T	T	C
3 isolates	M. abscessus subsp. abscessus	inducible (day 7)	Sequevar08	G	T	G	A	T	C	A	G	G	A	T	T	C
ERM001	M. abscessus subsp. abscessus	resistant	Sequevar08	G	T	G	A	T	C	A	G	G	A	T	T	C
ERM010	M. abscessus subsp. abscessus	resistant	Sequevar08	G	T	G	A	T	C	A	G	G	A	T	T	C
ERM086	M. abscessus subsp. abscessus	inducible (day 7)	Sequevar10	G	T	G	G	C	G	G	A	T	C	C	T	C
ERM057	M. abscessus subsp. abscessus	intermediate (day 3)	*Sequevar11*	G	T	G	A	C	G	G	A	T	C	C	T	T
ERM088	M. abscessus subsp. abscessus	inducible (day 7)	*Sequevar12*	G	T	G	G	C	G	G	A	T	C	C	T	T
ERM048	1bp from M. abscessus (16s rRNA)	inducible (day 7)	*Sequevar13*	G	T	G	A	C	G	G	T	G	C	C	T	C
2 isolates	M. abscessus subsp. abscessus	inducible (day 7)	*Sequevar14*	G	T	G	G	C	G	G	A	G	C	T	T	C
2 isolates	M. abscessus subsp. abscessus	inducible (day 10)	*Sequevar15*	G	T	G	A	T	G	A	G	G	A	T	T	T
26 isolates	M. abscessus subsp abscessus	inducible (day 7)	*Sequevar15*	G	T	G	A	T	G	A	G	G	A	T	T	T
2 isolates	M. abscessus subsp. abscessus	intermediate (day 3)	*Sequevar15*	G	T	G	A	T	G	A	G	G	A	T	T	T
ERM015	M. abscessus subsp. abscessus	resistant	*Sequevar15*	G	T	G	A	T	G	A	G	G	A	T	T	T
ERM040	M. abscessus subsp. bolletii	intermediate (day 3)	*SequevarB01*	G	T	G	A	C	G	G	G	G	C	C	T	C
ERM002	M. abscessus subsp. bolletii	inducible (day 7)	*SequevarB02*	G	T	G	A	C	G	G	G	G	C	C	T	C
ERM009	M. abscessus subsp. bolletii	resistant	*SequevarB03*	G	T	G	A	G	G	G	G	G	C	C	T	C
ERM017	1bp from M. abscessus (16s rRNA)	sensitive	truncated	N/A	N/A	N/A	N/A	N/A	N/A	N/A	N/A	N/A	N/A	N/A	N/A	N/A
ERM027	1bp from M. abscessus (16s rRNA)	sensitive	truncated	N/A	N/A	N/A	N/A	N/A	N/A	N/A	N/A	N/A	N/A	N/A	N/A	N/A
20 isolates	M. abscessus subsp. massiliense	sensitive	truncated	N/A	N/A	N/A	N/A	N/A	N/A	N/A	N/A	N/A	N/A	N/A	N/A	N/A

^1^A line list of data for individual strains can be found in S1 Table

^2^Sequevars noted in italics have not been previously published and have been named provisionally for the purpose of this study only. SNPs used to determine sequevar are presented in the fields directly to the right.

Two of the remaining *M*. *abscessus* subsp. *bolletii* isolates and the 1 remaining strain closely related to *M*. *abscessus* (1 bp away by 16 rRNA gene sequencing) showed inducible resistance at day 7. The last remaining *M*. *abscessus* supsp. *bolletii* strain was macrolide resistant upon initial interpretation at day 3. The *erm*(41) gene sequences for these 4 isolates did not cluster closely with the remaining *M*. *abscessus* subsp. *abscessus* isolates and were assigned provisional sequevar numbers for the purpose of this study only.

The 62 remaining *M*. *abscessus* subsp. *abscessus* isolates were separated into 3 sequevars with multiple strains, and 8 sequevars containing only a single strain. Five isolates were resistant upon initial read, 4 were intermediate upon initial read and resistant at day 7, 46 showed inducible resistance at day 7, 5 were inducible at day 10 and, 2 showed inducible resistance at day 14 (Table 1).

Sequevars 06, 08 and 15 contained the largest number of samples that did not contain the T28C mutation. These three sequevars were analyzed to determine if there was any correlation between the sequevar and the time to detection of inducible resistance using an Fischer’s Exact Test analysis in R. There was no statistically significant association between sequevar and time to detection of inducible resistance (p = 0.06). Further, the low number of samples in sequevar08 appear to drive the p value down giving the false impression of possible significance. There were not enough subsp. bolletii isolates available for this study to analyze if subspecies was correlated with a difference in time to detection of inducible resistance.

Overall, the majority of inducible resistance (87.2%) was detected by day 7, with an additional 9.1% of inducible resistance being detected at day 10. There were two isolates (3.6%) with MIC interpretations that did not reach “resistant” until day 14. Both of these isolates had an MIC of 8 μg/mL at day 14. These isolates belonged to Sequevar06 (predicted to have inducible resistance). Thirty-five isolates had interpretations of sensitive at day 14 of incubation. All of these isolates either belonged to Sequevar02 (containing a T28C mutation) or had truncated *erm*(41) genes. Of the 14 isolates incubated until day 21, only one had a 21 day read of intermediate, the rest remained sensitive. This isolate had a T28C mutation and was therefore expected to be sensitive.

## Discussion

The identification of a functional *erm*(41) gene in *Mycobacterium abscessus* isolates has called into question the clinical usefulness of macrolides in the treatment of these infections [7,15,16], although the CLSI suggests inclusion of macrolides in the case of inducible resistance due to a lack of sufficient alternate options, and emperical evidence showing some usefulness. Clinical management of cases is further confused by controversy in the subspeciation of the *M*. *abscessus* group of organisms, which were previously split into three subspecies, with two of those subspecies, *bolletii* and *massiliense* eventually being combined into subspecies *bolletii*, and the fact that many labs do not even differentiate the *M*. *abscessus/ chelonae* complex.

The CLSI has partially addressed concerns about the detection of inducible resistance by recommending a 14 day incubation period for non-pigmented rapidly growing NTM, but this recommendation was provisional pending the accumulation of further data [10]. Based on our findings, we would suggest that the addition of a day 7 read to the day 14 read of the panels would improve turn around times for the vast majority of *M*. *abscessus* isolates exhibiting inducible macrolide resistance, but the addition of a 21 day read provided no added value to the test. There were two strains that converted to resistance at day 14 from Sequevar06, and one strain that had an interpretation of intermediate at day 21(Sequevar02). Based on the trends for Sequevars02 and 06, the isolates from Sequevar02 would be expected to be sensitive and the isolates from Sequevar06 would be expected to exhibit inducible resistance. These results call into question the accuracy of interpretations at (or beyond) the day 14 mark as well as the clinical relevance of this level of “resistance”. Like Brown-Elliot et al, we suggest that isolates with a 14 day MIC of 4 or 8 μg/mL be interpreted with caution and be repeated, or preferably, have gene sequencing completed to aid in interpretation [14].

Previous evaluations of *erm(*41) gene sequences have suggested that *M*. *abscessus* subsp. *massiliense* isolates invariably have truncated *erm*(41) genes and this finding is replicated in this study [7,17]. There were also two strains that were mismatched from *M*. *abscessus* by 16s rRNA gene sequencing that had the truncated *erm*(41) gene evaluated in this study. Considering the fact that in mycobacteriology, even single nucleotide polymorphisms in the 16s rRNA gene sequences have been used to differentiate at the species level, it can be hard at times to assign organisms such as these an appropriate identification. Sequencing of the *erm*(41) gene can therefore be valuable in assisting with proper identification and clinical management of organisms such as these.

The number of *erm*(41) sequevars found in this study exceeded the numbers expected based on previously reported [14] that found only 7 sequevars in a sample of 85 *M*. *abscessus* subsp.*abscessus* isolates. This study identified 15 sequevars of subsp. *abscessus* isolates and 3 sequvars comprised of a single subsp. *bolletii* isolate each. Our study strains fell into 6 of the 10 previously published sequevars and 9 new sequevars. This points to further diversity than previously published within the *erm*(41) gene. The data presented here shows that, within each of the sequevars, diversity is present both in the presence of immediately detectable macrolide resistance (likely caused by mutations in the *rrl* gene [8,11]) and inducible macrolide resistance. Further, while the majority of all inducible resistance was detected by day 7 of incubation, there was also variability in this characteristic within individual sequevars. Therefore, clustering via sequevar gave us no additional information as to why some resistance is detected at day 7 and some not until day 14.

As in previous studies, truncated *erm*(41) genes are associated with *M*. *abscessus* subsp. *massiliense* and are always associated with macrolide sensitivity [7,14,17]. *M*. *abscessus* subsp. *bolletii* isolates can benefit from *erm*(41) gene sequencing for the prediction of inducible macrolide resistance. Two *M*. *abscessus* subsp. *abscessus* isolates with a T28C mutation showed intermediate MICs at day 14, along with one isolate that had an intermediate clarithromycin MIC at day 21. This could indicate that there is an alternate resistance mechanism at paly, but based on the trends within each of the sequevars represented, along with two strains that showed sensitivity upon repeat testing, it is more likely that bacterial contamination possibly affected the MIC or the efficacy of the clarithromycin in the MIC plates may wane after extended incubation, although this effect was not observed during the validation performed for this study. In any case, extending the incubation past 14 days appears to be of little value and *erm*(41) gene sequencing has a better potential for accurate detection of inducible macrolide resistance. Incubation times for the identification of macrolide resistance did not correlate with sequevar, therefore time to detection of inducible macrolide resistance is not associated with, and cannot be predicted by, *erm*(41) gene sequence.

## Conclusions

Ultimately, the results of this study lead us to recommend that the addition of a 7 day read of the broth microdiultion panels to the CLSI guidelines would improve turn around times for the majority (>80%) of *M*. *abscessus* isolates with inducible macrolide resistance [10]. Further, there is no additional value to adding a 21 day read to the Sensititre RAPMYCO panels. Finally, the routine differentiation of the members of the *M*. *abscessus/M*.*chelonae complex* down to the subspecies level, with the addition of of *erm*(41) gene sequencing for the detection of T28C mutations, would be an advantageous addition to phenotypic testing for the clinical management of *M*.*abscessus* cases. It is important to keep in mind, these results are related only to strains that are sensitive to macrolides after the standard 3-5day incubation period.

Based on the data presented here, presence of a truncated *erm*(41) gene in a macrolide-sensitive strain, accurately predicted the absence of inducible resistance in all cases, as did the presence of a T28C mutation. Any further characterization of the *erm*(41) did not appear to be predictive of resistance characteristics in this study. Our results suggest that molecular results would be sufficient to rule-in the use of a macrolide, although based on empirical evidence; macrolides may still be included in therapy in cases demonstrating inducible resistance. Further studies linking phenotypic and genotypic data from isolates demonstrating inducible resistance to patient outcomes will be required to determine if the molecular or phenotypic data correlates more closely with patient outcomes and if clinical recommendations should be changed. Assessment of whole genome sequence data from such cases may lead to insights into alternate causes for the continual “creeping-up” of the clarithromycin MIC in these isolates. Until such a time as that data is available, the use of both methodologies is recommended.

## Supporting Information

S1 Table Line list of subspeciation, days to detection of inducible macrolide resistance, *erm*(41) sequence and Sequevar data for study strains. (XLSX)
